# A rare case of congenital atresia of the left main coronary artery

**DOI:** 10.1016/j.radcr.2021.10.012

**Published:** 2021-11-16

**Authors:** Michaela Cellina, Letizia Di Meglio, Sara Marziali, Maurizio Cè, Giancarlo Oliva, Alessandro Visconti, Gianpaolo Carrafiello

**Affiliations:** a Radiology Department, ASST Fatebenefratelli Sacco, piazza Principessa Clotilde 3, 20121, Milan; b Postgraduation School in Radiodiagnostics, Università degli Studi di Milano, Via Festa del Perdono, 7, 20122 Milan, Italy; c General Management Department, ASST Fatebenefratelli Sacco, piazza Principessa Clotilde 3, 20121, Milan; d Radiology Department, Policlinico di Milano Ospedale Maggiore | Fondazione IRCCS Ca' Granda, Via Francesco Sforza, 35, 20122 Milan, Italy; e Dipartimento di Scienze della Salute, Università degli Studi di Milano, Via Festa del Perdono, 7, 20122 Milan, Italy

**Keywords:** Coronary artery disease, Coronary vessels, anatomy, Cross-sectional, Congenital abnormalities, Pathological conditions, Anatomical

## Abstract

Congenital atresia of the left main coronary artery is a rare coronary anomaly, in which the left coronary ostium is absent and the blood supply to the entire heart is provided by the right coronary artery. We show the images of the coronary CT angiography performed by a 56-year-old man, with evidence of this vascular abnormality. Coronary CT Angiography has gained a major role in coronary anomalies assessment, thank to high spatial and temporal resolution.

Congenital atresia of the left main coronary artery (CALM) is an extremely rare congenital anomaly of the coronary tree. It consists of the absence of the left coronary ostium, with right coronary artery supplying the entire heart [1,2]. The diagnosis of left main coronary artery (LMCA) is usually made in childhood due to the onset of symptoms, and it is only extremely rarely made in maturity.

Patients may be asymptomatic or present with syncope, dyspnea, myocardial infarction, or sudden death.

Surgical revascularization is advised in symptomatic individuals, although preventative surgical therapy is contentious in asymptomatic patients with LMCA.

Coronary congenital anomalies can be identified as incidental findings in patients undergoing coronary CT angiography (CCTA) for coronary artery disease.

Coronary angiography has been traditionally considered the gold standard for the study of coronary vessels; however, it is an invasive examination, associated with potential complications and high radiation exposure.

CCTA has gained a major role in coronary anomalies assessment, thank high spatial and temporal resolution, and lower radiation exposure. The possibility of different types of post-processing approaches, including multiplanar and three-dimensional reconstructions, allows a precise visualization of the anatomical configuration of the coronary tree that can be difficult to identify on 2D fluoroscopic images [3], [4], [5], [6].

We report a rare case of CALM, who underwent a CCTA for coronary artery disease suspicion.

## Case report

A 56-year-old man was referred to our radiology department for the execution of a CCTA due to persistent mild chest pain and a negative exercise testing. His past medical history was unremarkable. The examination was performed on a 256 multislice CT (Somatom Definition Flash, Siemens Forchheim, Germany) using automatic tube current modulation (CARE Dose 4D; Siemens Healthcare). The acquisition parameters, according to our Institution protocol, were as follow: reference tube current 320 mAs, tube voltage 100 kV, rotation time 0.28 second, collimation 128 × 0.6 mm, prospective ECG triggering. High Iodine concentration contrast medium (100 ML of Iomeron 400 mg I/mL, Bracco, Milan, Italy) was injected at 6 mL/s and bolus tracking technique was used to synchronize the start of the acquisition with contrast bolus arrival. The image dataset was then transferred to Syngo.via (Siemens Healthineers Global) for analysis and post-processing.

**Fig. 1:**
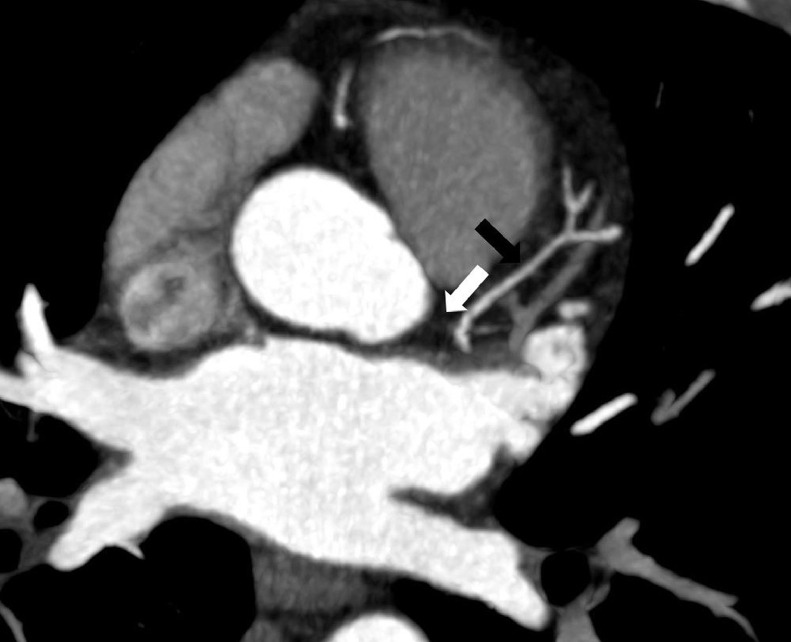
MIP reconstruction showing the absence of the LMCA (white arrow). The LDA is recognizable but diffusely thin (black arrow).

**Fig. 2:**
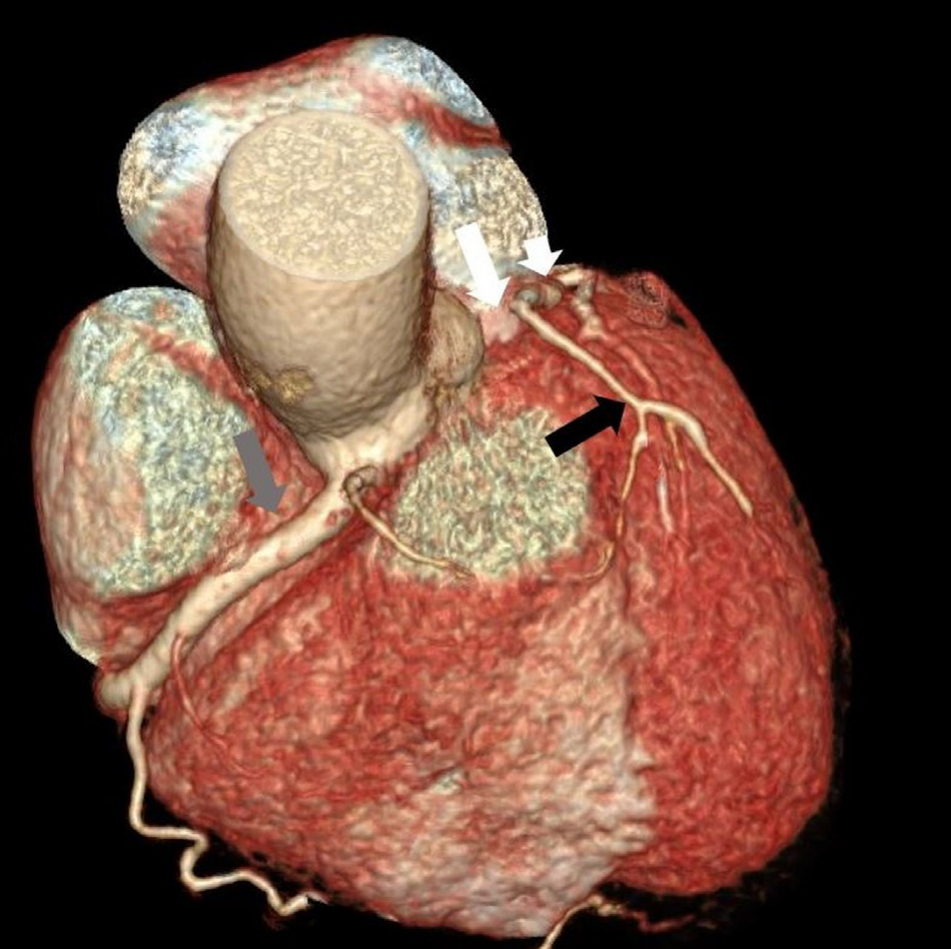
Three-dimensional reconstruction showing the absence of the left main coronary artery (white arrow). The LDA is recognizable, but thin (black arrow), as well as the proximal tract of the circumflex artery (short white arrow). The RCA is well evident, with a normal course (grey arrow).

**Fig. 3:**
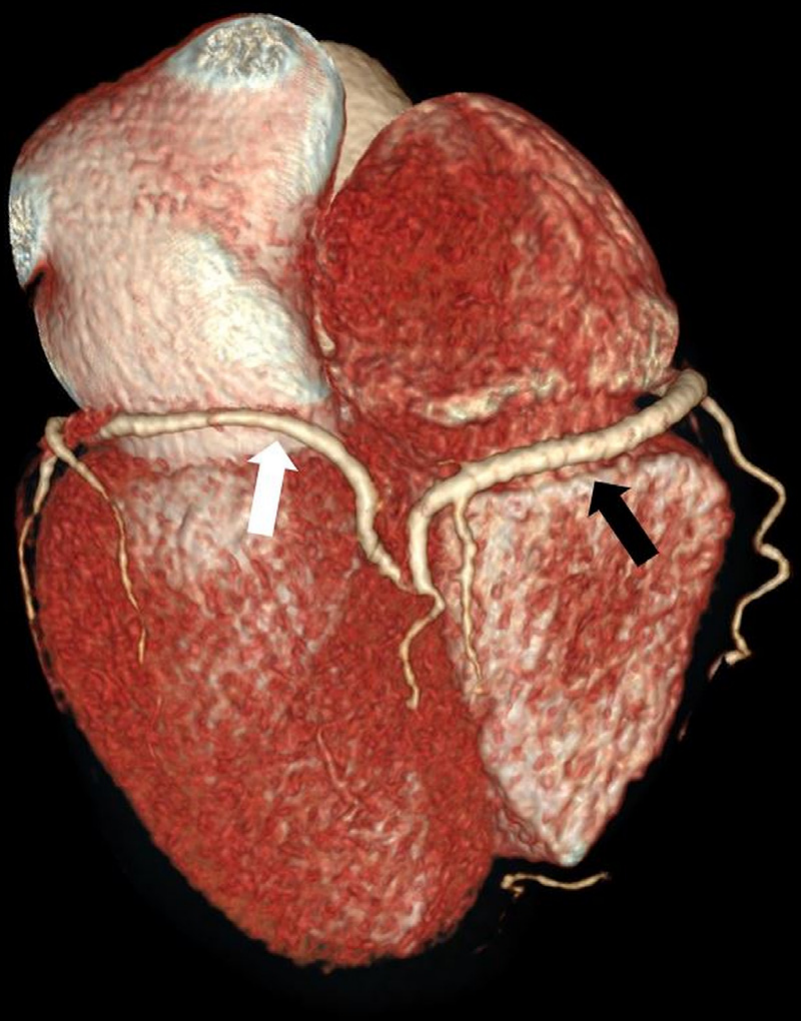
Three-dimensional reconstruction showing the connection between the RCA (black arrow) and the circumflex artery (white arrow).

**Fig. 4:**
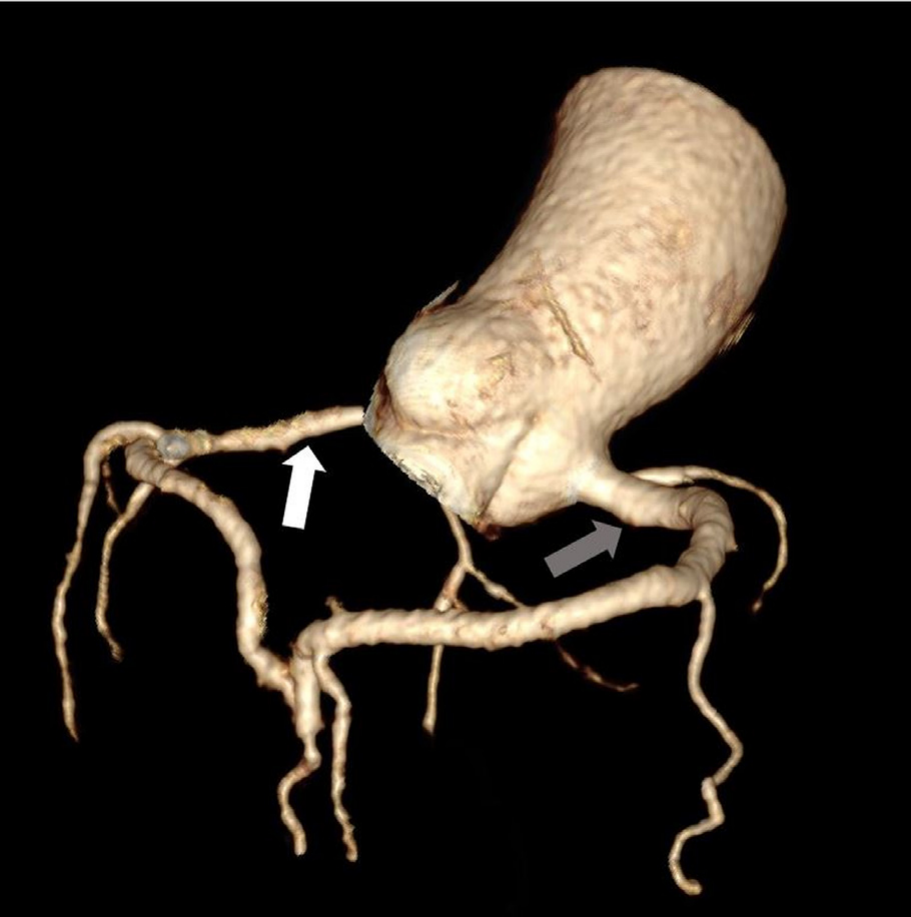
Three-dimensional evidence of the vascular ring creating by the connection between the RCA (grey arrow) and the circumflex artery (white arrow).

Images evaluation showed the absence of the left coronary ostium and of the LMCA (Fig. 1); the right coronary artery has regular course (RCA) (Fig. 2), and supplies the entire coronary circulation, through a connection with the circumflex artery at the posterior inter-ventricular sulcus, creating a vascular ring (Fig. 3, Fig. 4). Aortic valve had normal morphology. An anomalous vessel originates strictly adjacent to the RCA and contributes to the vascular supply of the left ventricular myocardium.

No coronary plaques were observed.

Multidisciplinary evaluation of the case decided for clinical follow-up.

## Discussion

CALM is an extremely rare condition. Patients usually present symptoms during infancy, youth, or adolescence, and this disorder is very seldom documented in adulthood and, when it is, it is an incidental finding.

It is characterized by the congenital absence of the LMCA and its ostium, with normally connected left anterior descending and circumflex arteries, which proximally end blindly [1,2]. Left anterior descending and circumflex arteries receive blood supply from the RCA through collateral circulation.

CALM seems to be caused by a defect of coronary development during the embryonic period; it is often an isolated coronary abnormality, but it may be also associated with other cardiac abnormalities, like pulmonary stenosis, ventricular septal defects, bicuspid aortic valve, and supravalvular aortic stenosis [7].

CALM belongs to the broad spectrum of congenital abnormalities of the coronary arteries that are extremely variable and include anomalies of their origin, the course of the epicardiac coronary branches, and distal connections [6,8].

CALM and ostial stenosis/atresia are both anomalies of the coronary origin, with a single coronary ostium. In CALM, a retrograde filling of the left coronary tree from the RCA takes place.

CALM can be identified as an incidental finding during coronary angiography or CCTA or associated to mild non-specific symptoms, however, this anatomical variant can manifest also with premature coronary disease, arrhythmias, syncope, myocardial infarction, or sudden cardiac death due to decreased myocardial perfusion [9]. In patients without inducible myocardial ischaemia, preventive surgical treatment is controversial.

In conclusion, we presented a case of a rare anatomical variant of the coronary tree, identified with CCTA. CCTA can be successfully applied for the study of congenital heart disease, in diagnosis and follow-up, thanks to the excellent visualization of the coronary arteries and anatomical relationships between vessels.

## Patient consent

Written informed consent was obtained from the subject in this study.
